# A golden orb-weaver spider (Araneae: Nephilidae: *Nephila*) from the Middle Jurassic of China

**DOI:** 10.1098/rsbl.2011.0228

**Published:** 2011-04-20

**Authors:** Paul A. Selden, ChungKun Shih, Dong Ren

**Affiliations:** 1 College of Life Sciences, Capital Normal University, Beijing 100048, People's Republic of China; 2 Paleontological Institute and Department of Geology, University of Kansas, Lawrence, KS 66045, USA; 3 Natural History Museum, London SW7 5BD, UK

**Keywords:** Daohugou, fossil, Inner Mongolia, Mesozoic

## Abstract

*Nephila* are large, conspicuous weavers of orb webs composed of golden silk, in tropical and subtropical regions. Nephilids have a sparse fossil record, the oldest described hitherto being *Cretaraneus vilaltae* from the Cretaceous of Spain. Five species from Neogene Dominican amber and one from the Eocene of Florissant, CO, USA, have been referred to the extant genus *Nephila*. Here, we report the largest known fossil spider, *Nephila jurassica* sp. nov., from Middle Jurassic (approx. 165 Ma) strata of Daohugou, Inner Mongolia, China. The new species extends the fossil record of the family by approximately 35 Ma and of the genus *Nephila* by approximately 130 Ma, making it the longest ranging spider genus known. Nephilidae originated somewhere on Pangaea, possibly the North China block, followed by dispersal almost worldwide before the break-up of the supercontinent later in the Mesozoic. The find suggests that the palaeoclimate was warm and humid at this time. This giant fossil orb-weaver provides evidence of predation on medium to large insects, well known from the Daohugou beds, and would have played an important role in the evolution of these insects.

## 1. Introduction

Nephilids are the largest web-weaving spiders alive today (body length up to 5 cm, leg span 15 cm) and are common and spectacular inhabitants of tropical and subtropical regions [1]. *Nephila* females weave among the largest orb webs known (up to 1.5 m in diameter), with distinctive golden silk. *Nephila* males are relatively diminutive compared with their conspecific females, providing an example of extreme sexual dimorphism [2–4]. Here, we describe the largest known fossil spider: a female *Nephila* from the Middle Jurassic of China.

Nephilidae contains 58 extant species in four genera in two subfamilies: Nephilinae (*Nephila*, *Herennia*, *Nephilengys*) and Clitaetrinae (*Clitaetra*) [5]. The Cenozoic record consists of three extinct genera in Paleogene (approx. 40 Ma) Baltic and Bitterfeld amber, five *Nephila* species from Neogene (approx. 16 Ma) Dominican amber [6] and *Nephila pennatipes* from the Eocene (approx. 34 Ma) of Florissant, CO, USA [7]. The Mesozoic record consists of *Cretaraneus vilaltae* from the Cretaceous (approx. 130 Ma) of El Montsec, Spain [8]. Supposed *Cretaraneus* from the Cretaceous of Brazil [9] and China [10] do not show the characters of the genus, and *Archaeometa nephilina* from the Carboniferous of England, described as resembling a *Nephila* [11], is probably not a spider [12]. The new species described here extends the fossil record of the family back by approximately 35 Ma, and the genus *Nephila* by approximately 130 Ma. All fossil nephilids apart from *N. pennatipes* are adult males and, in spite of their large size and hence better preservation potential in compression fossils, *Nephila jurassica* is only the second female fossil nephilid to be described.

## 2. Material and methods

The specimen comes from finely laminated, pale grey tuff in the Jiulongshan Formation near Daohugou Village, Wuhua Township, Ningcheng County, Inner Mongolia, China (41°19.532′ N, 119°14.589′ E). The Daohugou deposits yield a rich terrestrial biota, and their age is Middle Jurassic [13,14]. The specimen was studied, drawn and photographed dry under low-angle light, and under 70 per cent ethanol to enhance contrast. All measurements are in millimetres and were made from the photographs. The following species were studied for comparative purposes: *Nephila pennatipes* Scudder, 1885, holotype (Museum of Comparative Zoology no. 22596), Paleogene, CO, USA; *N. pilipes* (Fabricius, 1793), Recent, Taiwan; *N. clavipes* (Linnaeus, 1767), Recent, FL, USA.

## 3. Systematic palaeontology

Class: Araneae Clerck, 1757.

Family: Nephilidae Simon, 1894.

Genus: *Nephila* Leach, 1815.

Species: *Nephila jurassica* sp. nov.

*Derivation of name*: From Jurassic, the age of the fossil.

*Diagnosis*: *Nephila* with setal tufts (gaiters) in distal half of tibia 3 (in addition to tibiae 1, 2 and 4); opisthosoma ovoid, widest in anterior half, tapering posteriorly; epigyne nose shaped.

Holotype: CNU-ARA-NN2010008, College of Life Sciences, Capital Normal University, Beijing. No other specimens known.

**Figure 1.**
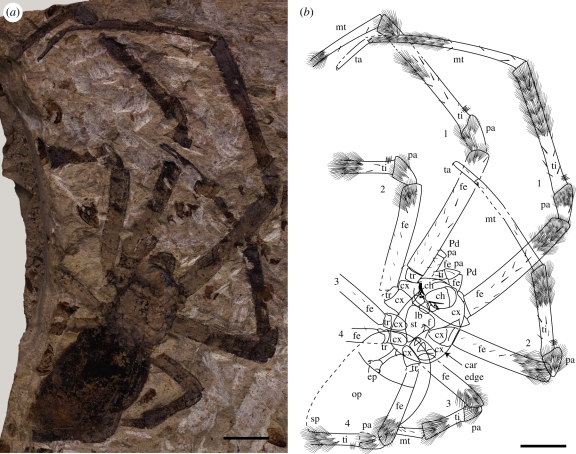
*Nephila jurassica* sp. nov. holotype (CNU-ARA-NN2010008). (*a*) Whole specimen, dry; (*b*) explanatory drawing to accompany (*a*); scale bar, 5 mm. Setal brushes and trichobothria diagrammatic. car, carapace; ch, chelicera; cx, coxa; ep, epigyne; f, fovea; fe, femur; lb, labium; mt, metatarsus; op, opisthosoma; pa, patella; Pd, pedipalp; sp, spinnerets; st, sternum; ta, tarsus; ti, tibia; tr, trochanter.

Description: Adult female. Specimen preserved dorsal-down in matrix so that left legs (in life) on right side of specimen (figure 1*a*,*b*). Ventral structures displaced to left relative to carapace, only edge of which is visible, but outline can be determined; longer than wide: length 9.31, width 6.83 (ratio 1.36); subrectangular, slightly bowed laterally, with anterior median projection, smooth lateral margins (no denticles); fovea shallow, recurved, with pair of recurved triangular grooves immediately posterior (forming an inverted W), 5.10 from anterior margin (i.e. slightly posterior to midpoint); carapace cuticle smooth with fine setae; eyes not visible. Sternum subtriangular, widest at anterior margin, not projecting between leg 4 coxae. Labium about as long as wide. Chelicerae revealed in low-angle light, superimposed on anterior part of carapace; short, stout, paturon directed ventrally in life (not correct), with curved fang and cluster of triangular teeth on cheliceral furrow; length 3.27. Parts of pedipalps visible in front of carapace: patella length 1.55.

**Figure 2.**
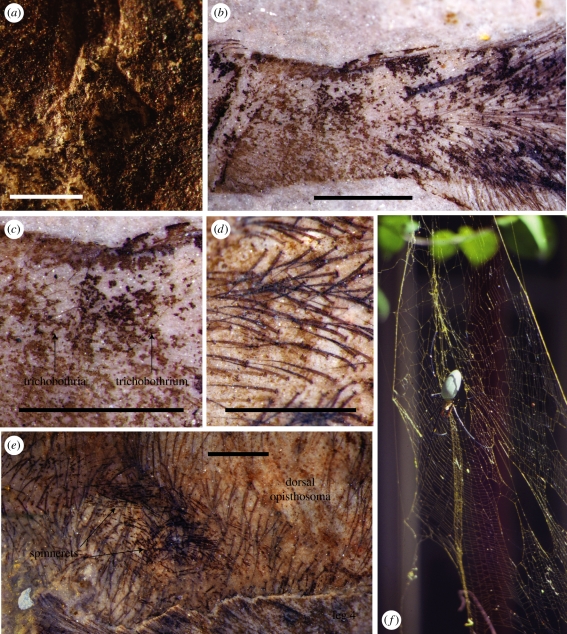
*Nephila jurassica* sp. nov. holotype. (*a*) Epigyne, (*b*) basal part of right leg 3 tibia showing setae, macrosetae, setal brush (right) and cluster of basal trichobothria, (*c*) close-up of (*b*) showing trichobothria, (*d*) coarse setae on dorsal opisthosoma, with a simple (i.e. not plumose) structure, (*e*) spinnerets and associated finer setae of the ventral surface in contrast to the coarser setae of the dorsal surface, and (*f*) *Nephila pilipes*, the giant golden orb-weaver and its web; photograph by P.A.S. at Cape Tribulation, Queensland, Australia. Scale bars, 1 mm. (*a*) Dry in low-angle light; (*b*), (*c*), (*d*) and (*e*) under ethanol.

Walking leg formula (longest to shortest) 1243; femora, tibiae and metatarsi of leg 1, and to a lesser extent 2, greatly elongated. Podomere lengths: leg 1 trochanter 1.25, femur 14.57, patella 3.75, tibia 15.39, metatarsus 16.92, tarsus 4.62; leg 2 femur 11.42, patella 3.25, tibia 9.56, metatarsus 11.49, tarsus 3.21; leg 3 femur 6.89, patella 2.10, tibia 4.95, metatarsus 5.24, tarsus 2.29; leg 4 femur 9.83, patella 2.56, tibia greater than or equal to 5.39. Macrosetae and fine covering of setae on all podomeres; setae smooth, not plumose (figure 2*d*). Trichobothria visible as fine, relatively short hairs crossing setation at high angle, emerging from distinct bothria. Femora with short, stout macrosetae ventrally, becoming longer distally, arranged in three rows proximally, two distally (fewer on shorter femur 3); no trichobothria; setal brush distally. Patellae with single lateral macroseta; setal brush on most of podomere. Tibiae with cluster of relatively short trichobothria (not feathery) proximally (figure 2*b*,*c*); prominent setal brush in distal half; scattered macrosetae. Metatarsi with setal brush in distal half; scattered macrosetae, cluster of at least three macrosetae at distal joint. Tarsi without setal brush; few, small macrosetae.

Opisthosoma left and posterior margins broken, but true margins probably lay close to preserved edges; longer than wide (length 15.36, width 9.50; length/width ratio 1.62), subovate, greatest width in anterior half and tapering gently posteriorly (not parallel-sided), densely clothed in fine setae. Low-angle light reveals compact, human nose-shaped epigyne (figure 2*a*) in median position on epigastric furrow; width 1.50. Posterior to epigyne, ventral opisthosomal cuticle fragmentary to absent, revealing coarser setation of dorsal surface (figure 2*d*), but patches of finer setae of ventral surface crossing coarser setae of dorsal surface represent spinnerets in subterminal position on opisthosoma (figure 2*e*).

## 4. Discussion

The large size of the female spider and the brushes of long setae on the ends of the tibiae are characteristics of the genus *Nephila*. These features also occur in some uloborids and tetragnathids, but these families show femoral trichobothria, and most tetragnathids have porrect chelicerae, which nephilids lack. The short trichobothria on the tibiae of the fossil are similar to those of modern *Nephila. Nephila jurassica* belongs to the subfamily Nephilinae, rather than Clitaetrinae which have a more circular opisthosoma, a narrow cephalic region, and longer femoral macrosetae [15,16]. Within Nephilinae, the fossil differs from *Herennia*, which has a lobed opisthosoma and warts on the carapace, and from *Nephilengys* which has conspicuous macrosetae on the carapace, only weakly tufted tibiae, longer trichobothria on the tibiae [17], and are mostly smaller than the fossil [1]. The opisthosoma of the fossil is long but not as elongate as in those extant *Nephila* in which it is more than twice as long as wide (e.g. *N. pilipes*) [18]. The body length of the fossil is comparable to that in *Nephila plumipes*, *N. clavipes* and *N. edulis*, species which also retain tibial setal tufts into adulthood. In general, smaller *Nephila* species and those with setal tufts in adults resolve more basally in most analyses [18]. It is likely that the fossil species lies within this basal group. While the large size of the fossil is suggestive of sexual dimorphism, this cannot be confirmed until a male is discovered; note, however, that all fossil male nephilids are of normal, small size.

Vicariance biogeographic analysis of extant nephilids suggested an age for the family of greater than 160 Ma [15], and molecular divergence estimates placed the origin of the genus as greater than 20 Ma, probably in tropical Asia or Africa [19], i.e. southern Pangaea. The new fossil evidence indicates an origin of the genus greater than or equal to 165 Ma, when Daohugou lay on the North China block in northern Pangaea [20]. In Jurassic times and earlier, animals could disperse across Pangaea, so an origin of *Nephila* anywhere on Pangaea was possible, probably followed by dispersal across the supercontinent before its break-up.

The palaeoclimate of Daohugou was considered to have been warm temperate based on the entomofauna [13]. Most modern *Nephila* live in tropical climates, and the few in subtropical/temperate conditions were considered to be derived [19]. So, the presence of *Nephila* at Daohugou could point to a warmer palaeoclimate than that suggested by the entomofauna, or that *N. jurassica* did, indeed, inhabit a warm temperate climate. If the latter, it is possible that *N. jurassica* is a derived lineage within the genus or, conversely, that the genus originated in a warm temperate climate and later radiated into the tropics. We note that the palaeoenvironment of Eocene Florissant, the provenance of *N. pennatipes*, has also been considered as warm temperate [21].

The sedimentology and palaeontology of the Daohugou beds suggest a fluviolacustrine palaeoenvironment with strong volcanic influence [13]. More than half of the insect families in the Daohugou biota are forest dwellers [13], as are Recent *Nephila*, which weave large, permanent orb webs of strong silk (figure 2*f*) to catch a wide variety of medium- to large-sized insects, mainly moths and beetles [22], but occasionally bats and birds [23] as by-catch. There are 18 orders of winged insects with high population counts among the Daohugou fossils [13]. It is likely that *N. jurassica* wove large, golden orb webs to catch medium- to large-sized insects in the Daohugou forests. Predation by these spiders would have played an important role in the natural selection of contemporaneous insects.
